# Long-Term Outcomes of Revisional Powered Endoscopic Dacryocystorhinostomy (EnDCR) with Intraoperative Application of Mitomycin C in Patients After Failed Laser-Assisted (LDCR) or External Dacryocystorhinostomy (ExDCR)

**DOI:** 10.3390/jcm14093116

**Published:** 2025-04-30

**Authors:** Michał Kinasz, Izabela Nowak-Gospodarowicz, Aleksandra Kinga Kicińska, Marek Rękas, Rafał Nowak

**Affiliations:** Department of Ophthalmology, Military Institute of Medicine, 04-141 Warsaw, Poland; mkinasz@wim.mil.pl (M.K.); inowak-gospodarowicz@wim.mil.pl (I.N.-G.); akicinska@wim.mil.pl (A.K.K.); mrekas@wim.mil.pl (M.R.)

**Keywords:** dacryocystorhinostomy, DCR, endoscopic dacryocystorhinostomy, nasolacrimal duct, lacrimal obstruction

## Abstract

**Background/Objectives:** The aim of the study was to evaluate the long-term outcomes of revisional powered endoscopic dacryocystorhinostomy (EnDCR) with the intraoperative application of Mitomycin C in patients after failed transcanalicular laser-assisted dacryocystorhinostomy (LDCR) or external dacryocystorhinostomy (ExDCR). **Methods:** This prospective, non-randomized, comparative, single-center clinical study was conducted between 2020 and 2023. The study included all patients presenting with epiphora graded ≥3 on the Munk scale (0–4) and confirmed ostium occlusion or significant narrowing on endoscopic examination following primary LDCR or ExDCR. All the participants underwent pre- and postoperative assessment using the Munk scale and fluorescein dye disappearance test (FDDT). The primary endpoints were ostium patency on irrigation and change in epiphora grade at 24-month follow-up. Secondary endpoints included changes in the FDDT results, endoscopic assessment of ostium patency, and ostium size. Outcomes were compared between the LDCR and ExDCR groups. **Results**: A total of 24 patients (mean age: 62 ± 19 years; range: 27–93 years) were included, with 12 cases after failed LDCR and 12 cases after failed ExDCR. The follow-up period ranged from 24 to 58 months. Significant improvement in epiphora was observed at the 24-month follow-up, both in the Munk scale (*p* < 0.001) and FDDT (*p* < 0.001). The overall anatomical and functional success rate was 95.8% (23/24). The mean time to recurrence was 63 weeks after ExDCR and 38 weeks after LDCR. Although there was a trend toward a longer symptom-free interval following ExDCR, the difference was not statistically significant (*p* = 0.231). **Conclusions**: Powered endoscopic DCR with intraoperative Mitomycin C application is an effective reoperative approach for managing recurrent lacrimal drainage obstruction following failed laser or external dacryocystorhinostomy.

## 1. Introduction

Dacryocystorhinostomy (DCR) is a surgical procedure that creates a fistula between the lacrimal sac and the nasal cavity, offering an alternative pathway for tear drainage in cases of nasolacrimal duct obstruction (NLDO). Modern surgical techniques include three main approaches: external (ExDCR), endoscopic endonasal (EnDCR), and transcanalicular laser-assisted (LDCR) procedures [1,2,3]. ExDCR is performed through a skin incision, with an osteotomy created in the lacrimal and maxillary bones to establish a connection between the lacrimal sac and the nasal mucosa. EnDCR is carried out under endoscopic guidance by opening the nasal mucosa over the lacrimal bone, creating an osteotomy, and incising the lacrimal sac to allow its flaps to heal against the nasal mucosa. In LDCR, a laser fiber is inserted through the canaliculus into the lacrimal sac, and laser pulses are used to create a fistula through the sac, lacrimal bone, and nasal mucosa.

For many years, ExDCR has been considered the “gold standard”, with reported success rates ranging from 70% to 95% [4,5,6,7]. However, its invasiveness and the presence of a postoperative scar have prompted the search for less burdensome alternatives, including LDCR and EnDCR [8,9].

LDCR is valued for its minimally invasive nature and shorter procedural time. However, its outcomes are highly variable, with the reported success rates ranging from 31% to 97%. This variability is primarily attributed to the small osteotomy size and the absence of the complete marsupialization of the lacrimal sac [10,11,12]. Conversely, EnDCR—initially viewed less favorably compared to the external approach [13]—has now evolved into a technique with comparable or superior efficacy. Reported success rates for EnDCR range from 84.4% to 100% [14,15,16,17,18].

The significant advancement of EnDCR can be largely credited to Wormald’s 2002 publication, which emphasized the importance of creating a wide osteotomy using powered instruments such as a microdebrider and diamond burr, achieving the complete marsupialization of the lacrimal sac [18,19,20].

The efficacy of DCR procedures depends on successfully creating and sustaining an epithelialized fistula between the lacrimal sac and the nasal mucosa, ensuring continuous tear drainage. The fibrotic closure of the fistula at the bony ostium is the most frequent cause of dacryocystorhinostomy failure [21]. In recent years, the benefit of applying Mitomycin C (MMC) at the dacryocystorhinostomy site to minimize scarring and therefore enhance surgical outcomes has been proven [22].

Despite the high overall effectiveness of DCR, a subset of patients continues to experience recurrent symptoms, particularly in revision cases. The aim of this study is to evaluate the long-term effectiveness of EnDCR as a treatment option in patients with recurrence following primary LDCR or ExDCR.

## 2. Materials and Methods

### 2.1. Study Design

This prospective, non-randomized, single-center clinical study was conducted between 2020 and 2023 at the tertiary ophthalmology department of the Military Institute of Medicine—National Research Institute in Warsaw, Poland. All the participants received an informed consent form, which they signed. The study adhered to the principles of the Declaration of Helsinki and received approval from the Bioethics Committee of the Military Chamber of Physicians (Resolution No. 8/WIM/2020) on 17 March 2020.

### 2.2. Study Group

The study included patients with epiphora who underwent powered endoscopic dacryocystorhinostomy (EnDCR) following failed primary transcanalicular laser-assisted DCR (LDCR) or external DCR (ExDCR). The inclusion criteria were as follows:

1. Adult patients (≥18 years) with general health permitting at least 12 months of follow-up;
2. Documented epiphora graded ≥3 on the Munk scale after previous DCR;
3. Endoscopic confirmation of ostium closure or significant narrowing.

The exclusion criteria included the following: age under 18 years, absence of significant epiphora (Munk scale < 3), major anatomical nasal abnormalities, post-traumatic obstruction, pregnancy or breastfeeding, and history of undergoing both types of DCR (e.g., initial LDCR followed by ExDCR, or vice versa).

All the patients presenting with epiphora underwent initial evaluation based on medical history, with an assessment of symptom duration (measured from the onset of epiphora after the previous surgery) and any prior history of inflammation or infection. This was followed by a comprehensive ophthalmic examination, including best-corrected visual acuity (BCVA) testing, slit-lamp biomicroscopy, and fundus examination to exclude alternative causes of epiphora.

Endoscopic examination of the nasal cavity was performed for all the patients, with special attention to the area of the previous surgical intervention (typically anterior to the middle turbinate). Lacrimal irrigation and probing were also conducted to determine the level and site of obstruction.

For each patient, the severity of epiphora was evaluated using the Munk scale with a grading range of 0–4 [23] and additionally assessed using the fluorescein dye disappearance test (FDDT), graded on a 0–3 scale [11,24,25].

### 2.3. Outcome Measures

The primary endpoints were ostium patency on lacrimal irrigation and the change in epiphora grade on the Munk scale at the 24-month follow-up.

Secondary endpoints included changes in the FDDT results, the endoscopic assessment of ostium patency, and ostium size. Clinical outcomes were compared between the LDCR and ExDCR subgroups.

### 2.4. Surgical Technique

All the procedures were performed under general anesthesia by a single oculoplastic surgeon with four years of experience in powered endoscopic DCR. A 4 mm endoscope with a 0° viewing angle was used to visualize the nasal cavity. Prior to the procedure, the nasal cavity was packed with gauze soaked in a solution of adrenaline and lidocaine in a 1:10,000 ratio to achieve mucosal decongestion and local anesthesia.

Nasal cavity preparation included the infiltration of the anterior portion of the middle turbinate base with 0.3 mL of a solution containing adrenaline and lidocaine in a 1:80,000 ratio. This was followed by repacking the nasal cavity with gauze soaked in a more concentrated adrenaline-lidocaine solution (1:1000 ratio).

An incision was then made in the nasal mucosa on the lateral nasal wall, just above the base of the middle turbinate. The incision began posteriorly and extended anteriorly, then continued vertically downward (up to two-thirds of the height of the middle turbinate), and finally proceeded horizontally backward, allowing access through the scar tissue.

The mucosal flap along with fibrotic tissue was elevated and removed using a microdebrider oscillating blade (Tricut^®^ Blade, REF 1884004HR, Medtronic, Minneapolis, MN, USA). Osteotomy was then performed by perforating the residual lacrimal bone and removing the remaining frontal process of the maxilla—previously spared during earlier procedures—using Kerrison rongeurs. The margins of the newly created ostium were refined with a 20-degree curved diamond burr (High-Speed Curved Diamond DCR Bur, REF 1882569HS, Medtronic, Minneapolis, MN, USA) ensuring smooth, regular edges to promote optimal mucosal healing and ostium patency. If the upper portion of the lacrimal sac remained, it was marsupialized by making a vertical incision and creating mucosal flaps, which were reflected outward and positioned to align with the edges of the newly created ostium, ensuring proper adhesion and long-term patency. If only scar tissue was encountered in the ostium area, the common canalicular opening was identified within the fibrotic tissue and subsequently marsupialized.

Lacrimal tract intubation was performed using Crawford silicone tubes (Crawford Intubation Set, REF S1.1270, FCI S.A.S., Paris, France). A circumostial injection of Mitomycin C (MMC) was administered to the lateral nasal wall as an intramucosal injection of 0.02% (0.2 mg/mL) MMC at four points (0.1 mL at each point) along the edges of the freshly created ostium. This was followed by the application of a sponge soaked in the same solution, placed on the edges of the created fistula for 3 min, as previously published elsewhere [19,26].

To complete the procedure, a combination ointment containing dexamethasone and an antibiotic (Maxitrol^®^, Alcon Inc., Fort Worth, TX, USA) was introduced into the nasal cavity. Nasal packing was then performed, and a long-acting steroid solution (Depo-Medrol^®^, Pfizer, New York, NY, USA) was instilled into the lacrimal ducts to reduce inflammation and prevent restenosis [26].

### 2.5. Postoperative Care

Postoperative treatment included the administration of combined antibiotic–steroid eye drops (Maxitrol^®^, Alcon Inc., Fort Worth, TX, USA) for a duration of four weeks. A nasal spray containing mometasone furoate (50 µg/dose) was prescribed for two weeks to reduce local inflammation. The Crawford stent was removed between 4 and 13 weeks postoperatively depending on the individual healing process.

Follow-up visits were scheduled on postoperative days 2–3, followed by fixed intervals at 2 weeks, and at 1, 3, 6, 12, and 24 months. Additional visits were arranged at any point during the follow-up period if complications arose or if patients reported concerning symptoms.

### 2.6. Statistical Analysis

Statistical analysis was conducted using the SPSS software (IBM Corp., 2012. IBM SPSS Statistics for Windows, Version 30.0, Armonk, NY, USA). Patient follow-up duration and data distribution characteristics were summarized using descriptive statistical methods. Comparisons of the time to recurrence of epiphora between the two groups (LDCR and ExDCR) were performed using the Mann–Whitney U test. Changes in the epiphora grade, assessed via the Munk scale, and changes in the FDDT scores before and after surgery were analyzed using the Wilcoxon signed-rank test.

Additionally, clinical parameters related to the healing process—such as endoscopic assessment of ostium size and results of lacrimal syringing—were analyzed descriptively in relation to the type of primary surgical procedure.

## 3. Results

A total of 24 patients (11 women and 13 men) were enrolled in the study:

- A total of 12 patients underwent external dacryocystorhinostomy (ExDCR);
- A total of 12 patients underwent laser-assisted dacryocystorhinostomy (LDCR).

**Figure 1:**
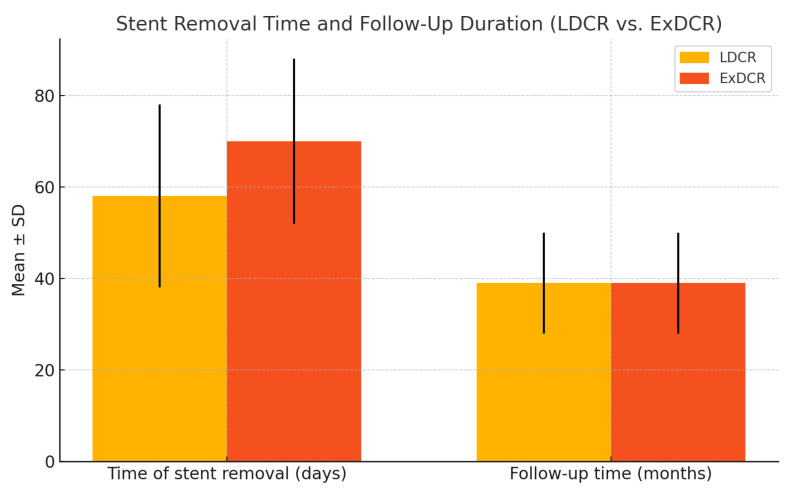
Time of stent removal and follow-up duration in revision EnDCR (LDCR vs. ExDCR).

The demographic and clinical characteristics of the study group are presented in Table 1. The follow-up period ranged from 24 to 58 months, with a mean of 39 ± 12 months (Figure 1). The mean time to recurrence was 63 weeks following ExDCR and 38 weeks following LDCR. Although there was a trend toward a longer asymptomatic period following ExDCR, the difference was not statistically significant (*p* = 0.231).

**Figure 2:**
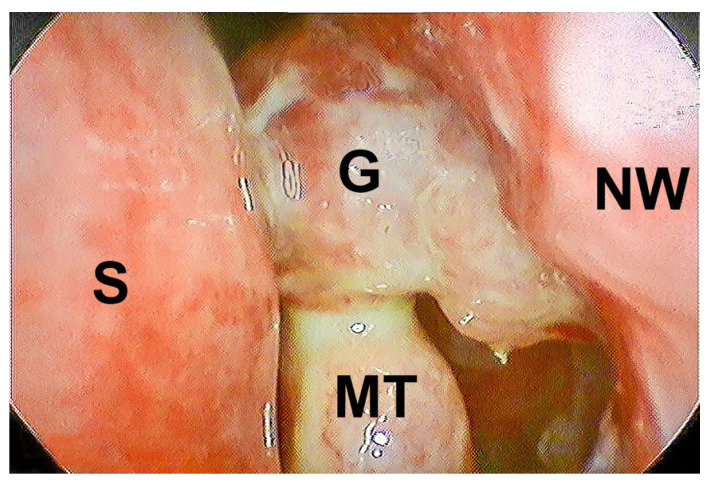
Ostium closure due to granulation (left side; G—granuloma at ostium; NW—nasal wall; S—septum; MT—middle turbinate).

Ostial occlusion or significant narrowing due to scarring was observed in 83.3% of all the cases. Granulation tissue formation accounted for dacryocystorhinostomy failure in 16.7% of the cases (Figure 2). Scarring was present in all the patients in the LDCR group (12/12; 100.0%) and in 8 patients in the ExDCR group (8/12; 66.7%). Granulation tissue was observed in 4 patients in the ExDCR group (4/12; 33.3%).

**Figure 3:**
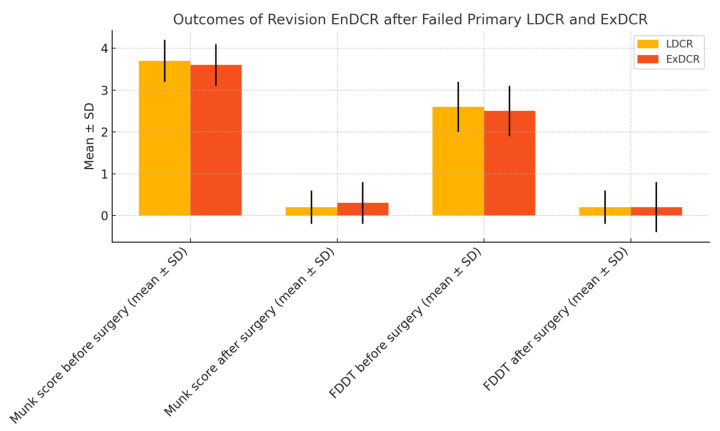
Outcomes of revision EnDCR after failed primary LDCR and ExDCR.

The statistical analysis using the Wilcoxon signed-rank test (Z = −4.378) revealed a significant reduction in epiphora grade postoperatively (*p* < 0.001). Similarly, a significant improvement in the fluorescein dye disappearance test (FDDT) was observed compared to the preoperative values (Z = −4.403, *p* < 0.001). All the patients demonstrated postoperative improvement, with no cases of clinical deterioration or unchanged results (Table 2, Figure 3).

**Figure 4:**
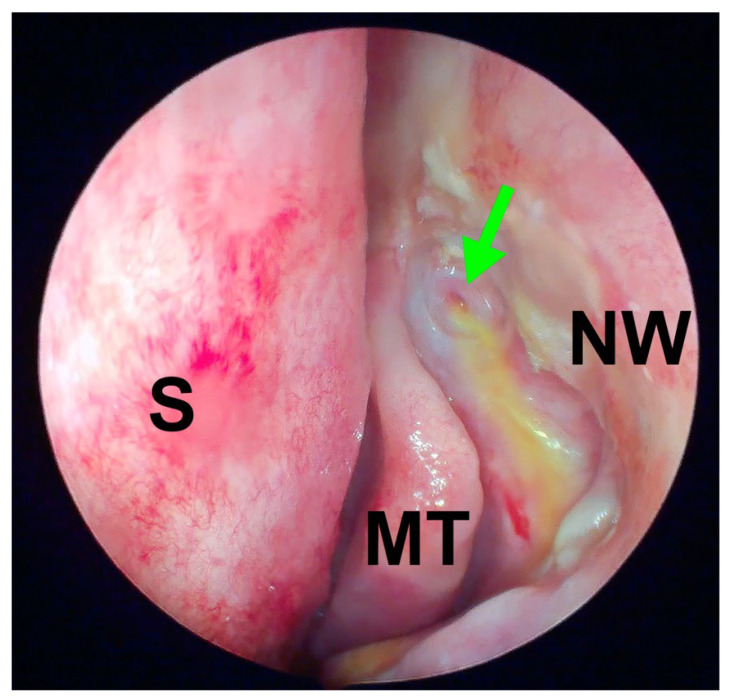
Patent ostium after revisional EnDCR due to previous failed ExDCR (left side; 6 weeks after surgery; green arrow—common canalicular opening; NW—nasal wall, MT—middle turbinate, S—septum; note yellowish fluorescein fluid coming out of common canalicular opening).

**Figure 5:**
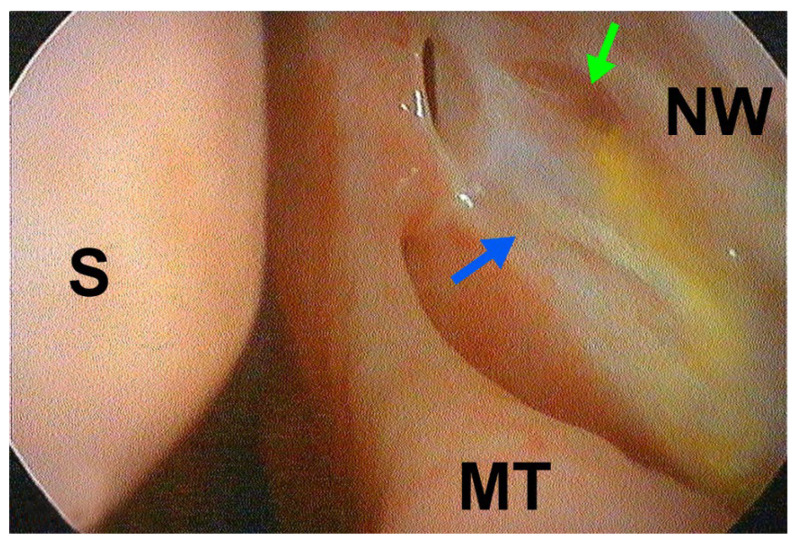
Healed patent ostium after revisional EnDCR due to previous failed LDCR, with ostium scarring being the cause of occlusion (left side; 16 weeks after surgery; green arrow—common canalicular opening, blue arrow—scarring at ostium edges; NW—nasal wall, MT—middle turbinate, S—septum; note yellowish fluorescein fluid coming out of common canalicular opening).

A data analysis indicated that the fistula patency assessed during follow-up (via lacrimal syringing), the size of the nasal ostium on endoscopic evaluation, and other healing-related clinical parameters (e.g., type of initial surgery, presence of comorbidities) did not significantly differ between the groups, suggesting a comparable healing process regardless of the previous surgical method (Figure 4 and Figure 5).

The overall anatomical and functional success rate was 95.8% (23 out of 24 patients), with only one case of symptom recurrence following reoperation.

## 4. Discussion

This study confirms the high anatomical and functional efficacy of powered endoscopic dacryocystorhinostomy (EnDCR) combined with intraoperative Mitomycin C (MMC), achieving a success rate of 95.8% (23/24). Unlike previous publications evaluating the outcomes of revision EnDCR, this is the first study to employ a prospective, comparative study design [27,28,29,30].

In the context of the existing literature, our findings support previous reports indicating that EnDCR is the preferred revision technique in cases of failed primary dacryocystorhinostomy (DCR) procedures [27,28,29,30,31,32]. The high effectiveness of EnDCR is largely attributable to superior visualization, which permits a wide osteotomy involving both the lacrimal bone and the frontal process of the maxilla, as well as the precise excision of scar tissue and adhesions [33]. Endoscopic guidance facilitates comprehensive marsupialization of the lacrimal sac—even in complex revision cases—thereby enhancing surgical outcomes.

This study further substantiates the limitations of laser-assisted DCR, which, despite being minimally invasive, is constrained by its small osteotomy size and inadequate marsupialization, factors that predispose to secondary closure of the fistula [11]. Although external DCR (ExDCR) demonstrates a high primary success rate, it remains a more invasive procedure associated with longer recovery times and a risk of visible scarring—an important consideration impacting patient satisfaction and cosmetic outcomes [34].

Our findings revealed that failure of the primary LDCR and ExDCR procedures performed externally was primarily due to insufficient osteotomy and inadequate marsupialization, resulting in postoperative scarring or granulation tissue formation within the ostium.

Agarwal et al. reported a revision EnDCR success rate of 94.5%, comparable to our results [27]. However, their cohort included patients with failed primary EnDCR and ExDCR. Korkut et al. noted a success rate of 84.1% for revision EnDCR, though the type of primary procedure was not specified [30]. Allon et al. documented a decline in success rate from 93.3% immediately after surgery to 71.1% at 2 years and to 68.9% at 5 years [29]. In our cohort, followed for 24 months, we did not observe a comparable decrease in success.

Importantly, none of the aforementioned studies utilized Mitomycin C during EnDCR. In contrast, Ali et al. demonstrated the safety and efficacy of MMC in preventing ostium closure following EnDCR [19]. In alignment with those findings, we employed MMC intraoperatively as described in prior protocols [26], which likely contributed to our high success rate.

This clinical study also evaluated the impact of surgery on the severity of epiphora, as assessed by the Munk score at 24-month follow-up. The complete absence of patients with worsening or unchanged epiphora scores at all the predetermined follow-up intervals (1, 3, 6, 12, and 24 months) further supports the high efficacy of revision EnDCR.

One female patient experienced a recurrence of epiphora at the six-month follow-up. An endoscopic evaluation revealed a large, patent nasal ostium, and the recurrence was attributed to postoperative corneal decompensation following cataract surgery rather than failure of the EnDCR.

The statistical analysis revealed no significant difference in the time to recurrence of epiphora between the patients who underwent primary LDCR versus ExDCR, as confirmed by both Student’s *t*-test and Mann–Whitney U test (*p* > 0.05). Although the difference did not reach statistical significance, the nearly twofold longer asymptomatic period following ExDCR may indicate a trend that could not be confirmed due to the limited group size. Additionally, the age distribution of the study population (ranging from 27 to 93 years, with a predominance of individuals over 60) showed no apparent impact on surgical outcomes, indicating uniform effectiveness across age groups.

The favorable outcomes achieved in this study are attributed to a combination of factors: excellent intraoperative visualization enabling the precise enlargement of the ostium with a microdebrider and diamond burr, and the use of Mitomycin C to reduce the risk of postoperative fibrosis and granulation [19].

Our results clearly support the high effectiveness of powered EnDCR in revision settings. Moreover, the absence of external scarring enhances the aesthetic and psychological acceptability of this approach from the patient’s perspective [13,32,35]. Considering the high success rate of EnDCR in patients with failed LDCR or ExDCR, this technique may be worth evaluating as a first-choice approach also in patients with primary nasolacrimal obstruction, especially given the known recurrence rates of other methods.

Like all clinical studies, this investigation has its limitations. The most notable is the relatively small sample size, which reflects the overall high success rate of dacryocystorhinostomy procedures in treating nasolacrimal duct obstruction. The limited number of cases also results from the nature of the study—it was prospective, comparative, and involved a long-term follow-up period. Although age heterogeneity did not appear to influence outcomes in this analysis, it may act as a confounding factor in broader populations with varying systemic health statuses. Nevertheless, subgroup analysis by age was not feasible due to the small sample size.

## 5. Conclusions

Powered endoscopic DCR combined with intraoperative Mitomycin C administration provided high long-term effectiveness in the treatment of patients with recurrence after laser or external dacryocystorhinostomy with no significant difference between these two groups. The findings from this study may contribute to the evolving consensus that endoscopic DCR should be considered the standard of care for primary surgical intervention in nasolacrimal duct obstruction. Broader, high-quality evidence from future research will be essential to refine surgical guidelines, standardize Mitomycin C protocols, and enhance personalized treatment approaches for patients with lacrimal drainage disorders.

## Figures and Tables

**Table 1:** The characteristics of the study group.

	LDCR Group	ExDCR Group
Age	62 ± 20	62 ± 20
Sex/Gender	F = 6, M = 6	F = 5, M = 7
Side	R = 6, L = 6	R = 5, L = 7
Munk score baseline	3.67 ± 0.49	3.58 ± 0.51

**Table 2:** Results.

	LDCR	ExDCR	*p*-Value
Munk score before surgery (mean ± SD)	3.67 ± 0.49	3.58 ± 0.51	*p* = 0.689
Munk score after surgery (mean ± SD)	0.17 ± 0.39	0.25 ± 0.62	*p* = 0.243
FDDT before surgery (mean ± SD)	2.67 ± 0.49	2.58 ± 0.51	*p* = 0.689
FDDT after surgery (mean ± SD)	0.17 ± 0.39	0.17 ± 0.58	*p* = 0.104
Time of lacrimal stent removal (days)	58 ± 20	70 ± 18	*p* = 0.482
Follow-up time (months)	39 ± 12	39 ± 12	*p* = 0.855

## Data Availability

The data presented in this study are available upon request from the corresponding author.

## Abbreviations

BCVA	best-corrected visual acuity
DCR	dacryocystorhinostomy
EnDCR	endoscopic dacryocystorhinostomy
ExDCR	external dacryocystorhinostomy
FDDT	fluorescein dye disappearance test
LDCR	laser-assisted dacryocystorhinostomy
MMC	Mitomycin C
NLDO	nasolacrimal duct obstruction
